# Tongue necrosis secondary to giant cell arteritis, successfully treated with tocilizumab: a case report

**DOI:** 10.1186/s12891-023-06465-z

**Published:** 2023-05-15

**Authors:** Young Min Cho, Lara El Khoury, Jonathan Paramo, Diane Michelle Horowitz, Jian Yi Li, Nina Kello

**Affiliations:** 1 Department of Rheumatology, Northwell Health, Donald and Barbara Zucker School of Medicine at Hofstra/Northwell, Long Island, NY USA; 2 Department of Pathology and Laboratory Medicine, North Shore University Hospital and Long Island Jewish Medical Center, Northwell Health, Donald and Barbara Zucker School of Medicine at Hofstra/Northwell, Long Island, NY USA

**Keywords:** Giant cell arteritis, Tongue necrosis, Tocilizumab, Refractory, Case report

## Abstract

**Background:**

Giant Cell Arteritis (GCA) is a large vessel vasculitis that most commonly presents with headache, scalp tenderness, jaw claudication, and vision changes. Various other, less common, manifestations have been reported in the literature such as scalp and tongue necrosis. Though most patients respond to corticosteroids, some cases of GCA are refractory to the high doses of corticosteroids.

**Case presentation:**

We present a 73-year-old female with GCA refractory to corticosteroids presenting with tongue necrosis. This patient significantly improved with a dose of tocilizumab, an IL-6 inhibitor.

**Conclusion:**

To the best of our knowledge, this is the first case report of a patient with refractory GCA presenting with tongue necrosis that had rapid improvement with tocilizumab. Prompt diagnosis and treatment can prevent severe outcomes such as tongue amputation in GCA patients with tongue necrosis, and tocilizumab may be effective for corticosteroid-refractory cases.

## Background

Giant cell arteritis (GCA) is a large vessel vasculitis. The clinical manifestations can involve systemic, neurologic, and ophthalmologic complications.

In GCA, the immature vascular dendritic cells (DCs) at the adventitial-medial interface of large vessels activate naïve CD4 T cells, which differentiate to Th1 and promotes the activation of macrophages, intramural infiltration of giant cell granuloma formation leading to hyperplasia of the intimal layer of the artery, and end-organ ischemia. The activated macrophages produce IL-6 and IL-1B, differentiating the naïve CD4 + T cells into Th17 effector cells. The clinical manifestations can be heterogenous and include but are not limited to temporal headaches, scalp tenderness, jaw claudication, sudden permanent visual loss, transient monocular or binocular vision impairment such as visual blurring, vision loss, or diplopia. Less common manifestations include, lingual, scalp, or lip necrosis, peripheral neuropathy, facial, submandibular swelling, and audiovestibular disturbance [1]. The lingual artery is the first branch of the external carotid artery and can manifest with edema, pallor, pain, and intermittent claudication [2]. The description is rare, but it can affect the older population and can be associated with more visual symptoms. The complications include lingual ischemia and necrosis. We present a case of a patient with GCA, who presented with tongue necrosis despite being on high doses of corticosteroids.

## Case presentation

A 73-year-old female, with a past medical history of a cerebral aneurysm, hypertension, and dyslipidemia presented on 11/25/2022 with sudden onset of headache, right jaw pain, and visual impairment for three days. The patient noticed intermittent spotty vision affecting the right eye and impacting her daily activities. She described having bilateral throbbing headaches at the temporal areas without relief after taking acetaminophen 650 mg daily.

**Fig. 1:**
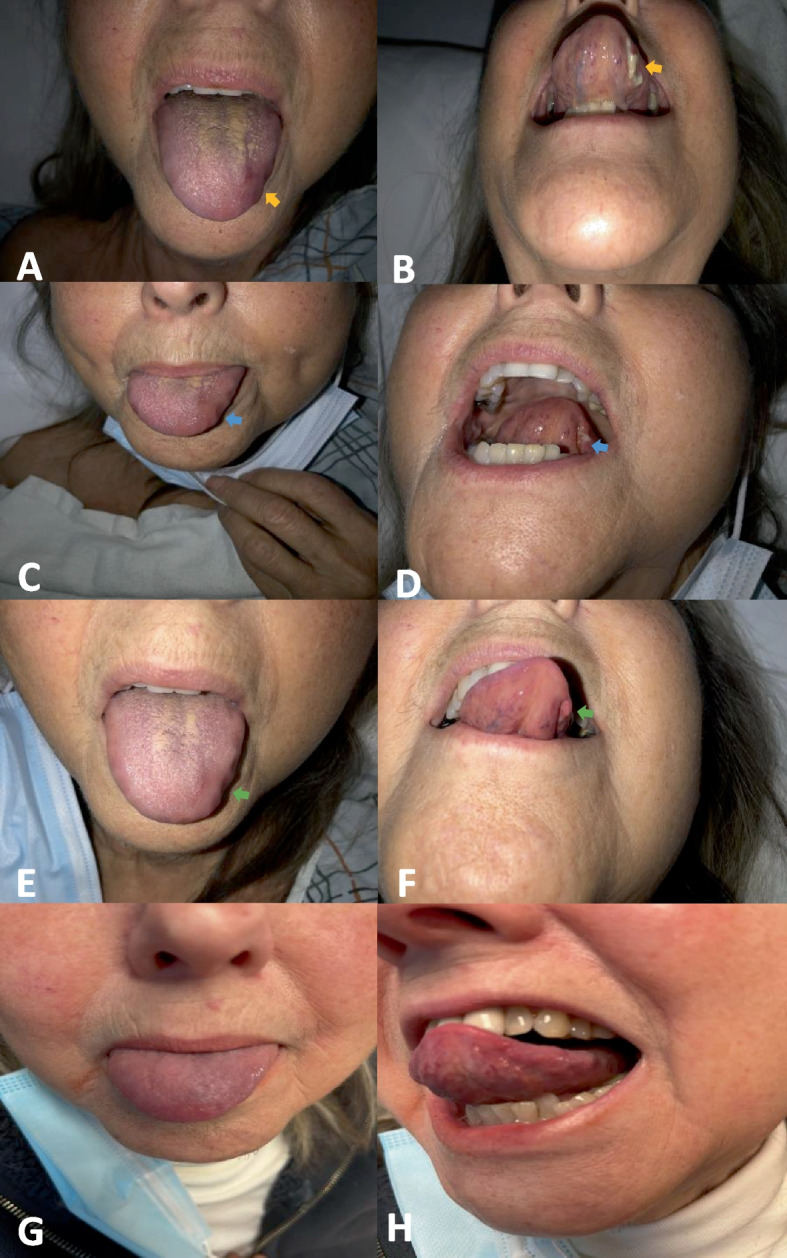
**A**, **B** yellow arrows indicating the lesion on the dorsal and ventral aspect of the tongue, respectively. **C**, **D** Day 1 following tocilizumab infusion. **E**, **F** Day 2 following tocilizumab infusion, noticeable improvement of lesions. **G**, **H** Two months later, the lesions in the tongue were completely healed

In the emergency room, her laboratory were white blood cell (WBC) 12.93 K/uL (NR 3.8–10.5 K/uL), hemoglobin 12.2 g/L (NR 11.5–15.5 g/dL), platelet 582 K/uL (150–400 K/uL), AND alkaline phosphatase 98 U/L (40–120 U/L). The inflammatory markers were elevated, erythrocyte sedimentation rate (ESR) 98 mm/hr (NR 0–29 mm/hr), and C-reactive protein (CRP) 150 mg/L (NR < 8 mg/L). A Computerized tomography angiogram (CTA) of the head and neck was negative. CTA of the chest and a transthoracic echocardiogram (TTE) were negative for large vessel involvement. Rheumatology was consulted to evaluate for possible GCA. Upon further history taking, the patient endorsed bilateral shoulder and hip pain with morning stiffness over the past three weeks, consistent with polymyalgia rheumatica (PMR). On physical exam, the patient had a diminished right temporal artery pulse compared to the left. A funduscopic exam by an ophthalmologist was remarkable for sharp and mild pallor optic nerve without edema or hemorrhage. The patient was started on intravenous (IV) methylprednisolone 1 g daily for three days. On the second day of admission, the patient endorsed significant improvement in her vision, jaw claudication, and headache. Subsequently, on 11/30/2022 the patient underwent bilateral temporal artery biopsies. On day four, the patient was transitioned to prednisone 60 mg daily, which was continued upon discharge. The pathology report of the temporal artery biopsies showed temporal arteritis on Hematoxylin and Eosin, trichrome and elastic stains (Fig. 1).

**Fig. 2:**
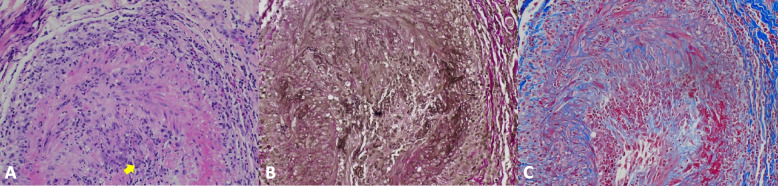
Temporal artery biopsy results. **A** Hematoxylin and Eosin stain showing inflammatory cells (predominant lymphocytes and occasional eosinophils) in intima, media and adventitia and rare multinucleated giant cells in intima indicated by yellow arrow, a characteristic feature of temporal arteritis. **B** Elastic stain showing the fragmentation, distortion, and lack of continuity of the internal elastic lamina. **C** Trichrome stain showing damage of the internal elastic lamina and media, and occlusion of lumen

**Fig. 3:**
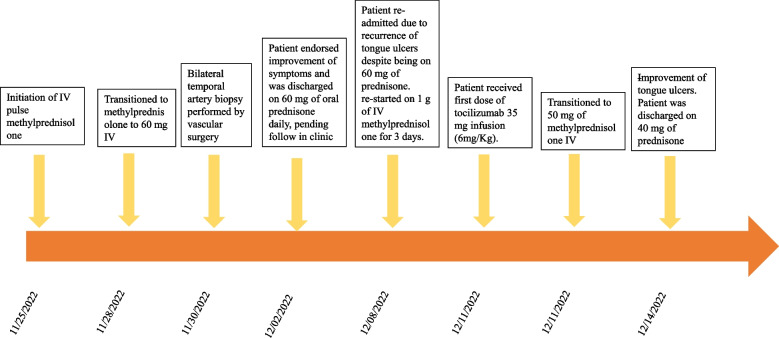
Timeline of case presentation description

**Fig. 4:**
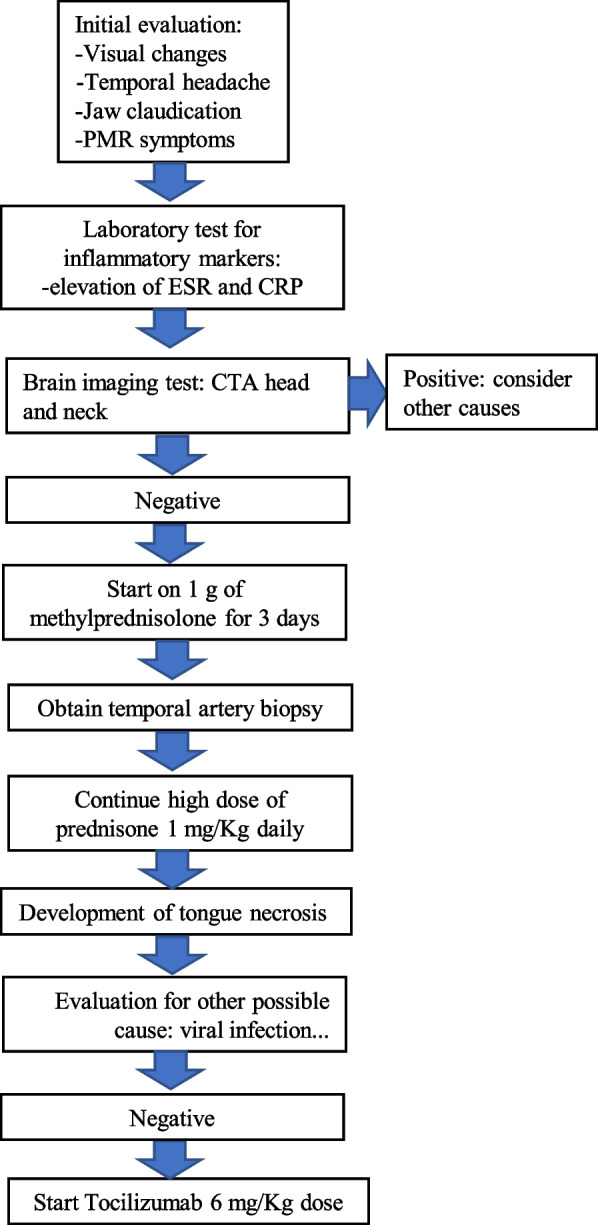
Scheme of management plan

Within a week of discharge, on 12/8/2022 the patient reported new onset tongue swelling and pain as well as worsening jaw claudication. Upon examination, the patient was noted to have edema of the left side of the tongue and an ulcerative lesion on the ventral portion of the tongue (Fig. 2). The patient reported compliance with taking prednisone 60 mg daily. Her inflammatory markers on admission were remarkable for an ESR of 53 mm/hr (NR 0–29 mm/hr), and CRP of 4.9 mg/L (NR < 8 mg/L). The patient was restarted on 1 g of IV methylprednisolone in the emergency room and was admitted for further evaluation. The ulcerative lesion was swabbed to evaluate for a viral infection such as herpes simplex, herpes zoster, and COVID-19, which came back negative [3]. On 12/11/2022, the patient received 350 mg (6 mg/Kg) intravenous tocilizumab with the plan to repeat in 4 weeks. Four days after the infusion of tocilizumab, the patient noticed a significant improvement in jaw pain and tongue swelling (Fig. 2). On 12/14/2022, the patient was able to tolerate oral intake and was discharged on prednisone 40 mg daily. Since her discharge, the tongue necrosis has resolved, and she has been continued on monthly tocilizumab infusions and she is on tapering doses of prednisone (Figs. 3 and 4).

## Discussion and conclusions

Tongue necrosis is an uncommon clinical manifestation of GCA, manifesting with tongue ulcers, edema, pallor, and pain. The tongue’s main blood supply comes from the lingual artery, a branch of the external carotid artery [4]. Tongue necrosis as a manifestation of GCA was first reported in 1959, and 21 cases have been reported since then [5]. The findings of our literature review are detailed in Table 1. Based on our findings, the average age of the patients was 77 ± 7.45 years, and the female-male ratio of 17:4. 14 out of 21 cases reported headache with jaw pain, and 77 cases with visual involvement. Tongue manifestations included pain, pallor, cyanosis, and necrosis. Among 21 cases, only 1 case had extra-cranial large vessel vasculitis which showed in the fluorodeoxyglucose (FDG)-PET without clinical manifestations. The diagnosis of GCA was based on clinical manifestations, elevated inflammatory markers, temporal artery biopsies in 16 cases and 1 case proven by temporal artery ultrasound alone. Most patients responded well to corticosteroids (doses ranging from prednisone 1 mg/kg to methylprednisolone 1000 mg) and three required tongue amputations. One case reported a good response to concomitant use of corticosteroids and tocilizumab as first line therapy due to extensive involvement of the arterial bed based on abnormal ultrasound findings of the deep lingual artery that included halo sign, increased intima media thickness and markedly reduced blood flow [6].

**Table 1:** Case reports and case series of GCA with tongue involvement

Authors	Date of publication	Age of patient	Clinical manifestations	Laboratory	Biopsy proven GCA	Treatment received	Extra-cranial large vessel vasculitis	Outcomes
Zaragoza et al. [7]	1/31/2015	68-year-old female	Moderate headache, swelling of the neck, and tongue edema with discoloration	ESR: 55 mm/hr CRP: 130 mg/L	Yes, bilateral temporal biopsies	1 mg/Kg corticosteroid	No	Extensive necrosis of the tongue that progressed to self-amputation
Sobrinho et al. [8]	3/23/2017	85-year-old male	Frontotemporal headache, jaw pain, tongue swelling, pain, and ulcers	ESR: 120 mm/hr CRP: 172 mg/L	Yes, temporal artery biopsy	1 mg/Kg corticosteroid and continue with methotrexate 10 mg weekly	No	Improvement of the tongue necrosis without further progression in the lesion
DeBord et al. [2]	5/24/2019	77-year-old female	Tongue pain with ulcers, odynophagia, and dehydration	ESR: 65 mm/hr	Yes, temporal artery biopsy	High dose of steroid	No	Tongue necrosis resolved
Jennings et al. [9]	12/2011	79-year-old female	Sore throat, bilateral occipital neck pain, dental pain, jaw claudication, and tongue necrosis	ESR: 75 mm/hr	Yes, temporal artery biopsy	High dose of steroid	No	Tongue completely auto-amputating
Oliver et al. [10]	5/23/2022	86-year-old female	Headache, right amaurosis, jaw claudication, lingual burning sensation with subsequent ulceration	ESR: 82 mm/hr	No	40 mg/day prednisone for 30 days	No	Tongue necrosis improvement
Bobinskas et al. [11]	5/18/2015	65-year-old female	Paresthesia, pallor, cyanosis of the tongue	ESR: 42 mm/hr	Yes, temporal biopsy	High dose of steroid	No	Tongue lesions improved
Burg et al. [6]	3/20/2021	78-year-old female	Visual change, bilateral temporal headache, thrusting bilateral pain in the mandible, tongue lesions, polymyalgia symptoms	ESR: 75 mm/hr CRP: 71 mg/l	No biopsy proven but ultrasound showed halo-sign for diagnostic purpose	Methylprednisolone 500 mg daily for 5 days then prednisone 60 mg daily and 162 mg tocilizumab weekly	No	Tongue lesion improved
Lobato-Berezo et al. [12]	9/26/2014	74-year-old female	Headache, blurred vision in her left eye, jaw pain, tongue pain and necrosis	No reported	Yes, right temporal artery biopsy	1 mg/Kg oral prednisone	No	Tongue lesion improved
Fongaufier et al. [13]	2018	66-year-old male	Bilateral headaches, tongue tenderness, jaw claudication, intermittent binocular diplopia with transient amaurosis	CRP: 120 mg/L	Yes, temporal artery biopsy	1.5 mg/Kg for 3 days	No	Emergent surgery for tongue resection with subsequent improvement
Tseytlin et al. [14]	1/2019	87-year-old female	Polymyalgia rheumatica symptoms, left jaw pain, dysphagia, tongue pain and ulceration	ESR: 68 mm/hr	Yes, temporal artery biopsy	50 mg of prednisone and methotrexate	No	Tongue lesion improved
Jalaledin et al. [15]	10/14/2022	76-year-old female	Bilateral headache, fatigue, weight loss, jaw pain, sudden right eye vision loss, and tongue ulcer	CRP: 159 mg/L	Yes, temporal artery biopsy	methylprednisolone IV 1 g for 3 days	No	Tongue lesion improved
Donaldson et al. [16]	6/1/2015	61-year-old female	Weight loss, myalgias, headache, jaw pain, and tongue lesions	CRP: 288 mg/L	No, proven biopsy. CT chest showed thickening of the wall of the arch of the aorta	Steroid (dose not specified)	No	Tongue lesion improved
Benedetti et al. [17]	4/14/2020	77-year-old male	Mental status change, dysarthria, right tongue discoloration	ESR: 80 mm/hr CRP: 390 mg/L	Yes, right occipital artery	1 g IV methylprednisolone	No	Tongue lesion improved
Biebl et al. [18]	2004	79-year-old female	Left eye visual change, headache, abdominal pain, and togue necrosis		Yes, right temporal artery biopsy	Prednisolone 100 mg daily and azathioprine 100 daily		Tongue necrosis stop spreading
Grant et al. [19]	2013	79-year-old female	Sudden vision loss of the left eye, occipital headache, ear, and jaw claudication, large necrotic tongue lesion	ESR: 68 mm/hr CRP: 150 mg/L	No	500 mg of IV methylprednisolone for 3 days and continue high dose of steroid	No	Tongue lesions healed well
Rose et al. [20]	11/16/2021	81-year-old female	Occipital headache, right eye vision loss, stroke, tongue necrosis	ESR: 102 mm/hr CRP: 163 mg/L	Yes, temporal artery biopsy	1 g of IV methylprednisolone for 3 days and continue with 60 mg of oral prednisone	No	Tongue ulcer started after the pulse of steroid with subsequent improvement after continuing high dose of steroid
Dos Reis et al. [21]	1/10/2021	91-year-old female	Tongue ulcer, jaw numbness, hearing loss, visual impairment, swallowing difficulty, and mild headache episode	ESR: 22 mm/hr CRP: 84 mg/L	No	1 mg/Kg prednisone for 4 weeks	No	Tongue necrosis improved
Kumarasinghe et al. [22]	11/19/2012	74-year-old female	Tongue lesion with numbness, and pain, mild headaches, jaw pain on chewing	ESR: 103 mm/hr CRP: 37 mg/l	Yes, temporal artery biopsy	40 mg of prednisolone daily, initial loading dose 300 mg with subsequent 75 mg daily		Tongue necrosis improved
Husein-ElAhmed et al. [23]	1/8/2010	76-year-old female	Painful tongue necrosis and swelling	ESR: 87 mm/hr	Yes, temporal artery biopsy	High dose of corticosteroid^a^	No	Tongue necrosis improved
Brodmann et al. [24]	3/10/2009	a. 81-year-old male b. 79-year-old female	a. Tongue ulcer b. Right sudden visual loss, headache, right temporal headache, jaw claudication	a. ESR: 52 mm/hr b. ESR 70 mm/hr	a. Yes, temporal artery biopsy b. Yes, temporal artery biopsy	a. High dose of steroid^a^ b. High dose of steroid^a^	a. No b. Yes, FDG-PET uptake in both subclavian arteries	a. Tongue necrosis improved b. Tongue necrosis improved

^a^The authors did not specify the dose of steroid

When evaluating tongue necrosis, several important differential diagnoses need to be considered, including malignancy (carcinoma, lymphoma, and sarcoma); adverse effects from medications (vasopressin, chemotherapy, and ergotamine); radiation therapy; cardiovascular etiologies (hemorrhage, embolism, and cardiac arrest); infection (syphilis, tuberculosis. Herpes); and systemic vasculitis (giant cell arteritis, and ANCA positive vasculitis) [7]. In our case, the patient tested negative for tuberculosis, and she was not taking any culprit medications. Therefore, due to ongoing high dose steroid therapy, herpetic infection was high in our differential, but was ruled out with a negative PCR from the lesion.

Glucocorticoid resistance is considered in patients with GCA whose reduction of glucocorticoids under 5 mg/day prednisolone equivalent is not possible [25]. Multiple studies have described methotrexate (MTX) as a possible steroid sparing agent and an option to treat GCA refractory to glucocorticoids. However, clinical trials have failed to show compelling outcomes, albeit most studies were done with lower doses of MTX [26]. There has not been enough data for azathioprine, and the only trial that demonstrated remission of disease under 5 mg of prednisone was not statistically significant [27]. The use of cyclophosphamide was reported in a case series, but no randomized controlled trials were conducted in GCA [28]. Eight of 10 cases achieved remission, but only in combination with an additional steroid-sparing agent such as MTX, AZA, or mycophenolate (MMF). TNF-inhibitors such as infliximab or etanercept showed no superiority in reducing corticosteroid dose [29, 30].

After the publication of the GiACTA trial by Stone et al., tocilizumab was approved as a steroid agent for GCA by the Food and Drug Administration (FDA) in 2017. In the trial, the GCA patients on tocilizumab plus a 26-week prednisone taper showed superiority to maintain corticosteroid-free remission compared to those on 52 week and 26-week prednisone tapers plus placebo [31].

In our case, the patient showed rapid improvement after receiving a single dose of IV tocilizumab. With the exception of a study by Burg et al., which employed tocilizumab as first line treatment, there have been no reports or case studies on the management of corticosteroid-refractory lingual necrosis associated with giant cell arteritis (GCA) using tocilizumab. The IL-6 secreted by Th17 cells has an essential role in GCA patients refractory to glucocorticoids [32], likely explaining our patient's positive outcome to tocilizumab.

In conclusion, GCA has a heterogeneous presentation, and one of the atypical manifestations reported is tongue necrosis. Rapid diagnosis and treatment can prevent dire outcomes such as tongue amputation. IL-6 plays an essential role in GCA pathogenesis, and its inhibition can be used as a treatment for GCA refractory to glucocorticoid therapy. To the best of our knowledge, this is the first case report of tongue necrosis refractory to corticosteroids successfully treated with tocilizumab. The novelty of this case report highlights the importance of keeping a high index of suspicion for potential complications that can develop during the course of the disease despite the corticosteroid treatment. The case raises the highly debated question of starting tocilizumab as the first line agent up front, proposing that it could potentially decrease the risk of severe complications.

## Data Availability

The datasets used during the current study are available from the corresponding author on reasonable request.

## Abbreviations

GCA: Giant cell arteritis
DCs: Dendritic cells
FDG-PET: Fluorodeoxyglucose-positron emission tomography
MTX: Methotrexate
AZA: Azathioprine
MMF: Mycophenolate
